# Techniques for navigating postsurgical adhesions: Insights into mechanisms and future directions

**DOI:** 10.1002/btm2.10565

**Published:** 2023-06-26

**Authors:** Jiahui Chen, Xiaoqi Tang, Ziyu Wang, Arielle Perez, Benjamin Yao, Ke Huang, Yang Zhang, Martin W. King

**Affiliations:** ^1^ Department of Textile Engineering, Chemistry and Science North Carolina State University Raleigh North Carolina USA; ^2^ UNC School of Medicine Department of Surgery University of North Carolina Chapel Hill North Carolina USA; ^3^ Montefiore Medical Center Department of Obstetrics & Gynecology & Women's Health Services Montefiore Medical Center Bronx New York USA; ^4^ Joint Department of Biomedical Engineering North Carolina State University & University of North Carolina at Chapel Hill Raleigh North Carolina USA; ^5^ Department of Molecular Biomedical Sciences North Carolina State University Raleigh North Carolina United States; ^6^ College of Textiles, Donghua University Shanghai Songjiang China

**Keywords:** abdominal adhesion, adhesion prevention, anti‐adhesion strategy, anti‐inflammatory, fibrinolytic agent, physical barrier, postsurgical adhesion

## Abstract

Postsurgical adhesions are a common complication of surgical procedures that can lead to postoperative pain, bowel obstruction, infertility, as well as complications with future procedures. Several agents have been developed to prevent adhesion formation, such as barriers, anti‐inflammatory and fibrinolytic agents. The Food and Drug Administration (FDA) has approved the use of physical barrier agents, but they have been associated with conflicting clinical studies and controversy in the clinical utilization of anti‐adhesion barriers. In this review, we summarize the human anatomy of the peritoneum, the pathophysiology of adhesion formation, the current prevention agents, as well as the current research progress on adhesion prevention. The early cellular events starting with injured mesothelial cells and incorporating macrophage response have recently been found to be associated with adhesion formation. This may provide the key component for developing future adhesion prevention methods. The current use of physical barriers to separate tissues, such as Seprafilm®, composed of hyaluronic acid and carboxymethylcellulose, can only reduce the risk of adhesion formation at the end stage. Other anti‐inflammatory or fibrinolytic agents for preventing adhesions have only been studied within the context of current research models, which is limited by the lack of in‐vitro model systems as well as in‐depth study of in‐vivo models to evaluate the efficiency of anti‐adhesion agents. In addition, we explore emerging therapies, such as gene therapy and stem cell‐based approaches, that may offer new strategies for preventing adhesion formation. In conclusion, anti‐adhesion agents represent a promising approach for reducing the burden of adhesion‐related complications in surgical patients. Further research is needed to optimize their use and develop new therapies for this challenging clinical problem.

## 1 INTRODUCTION

**FIGURE 1:**
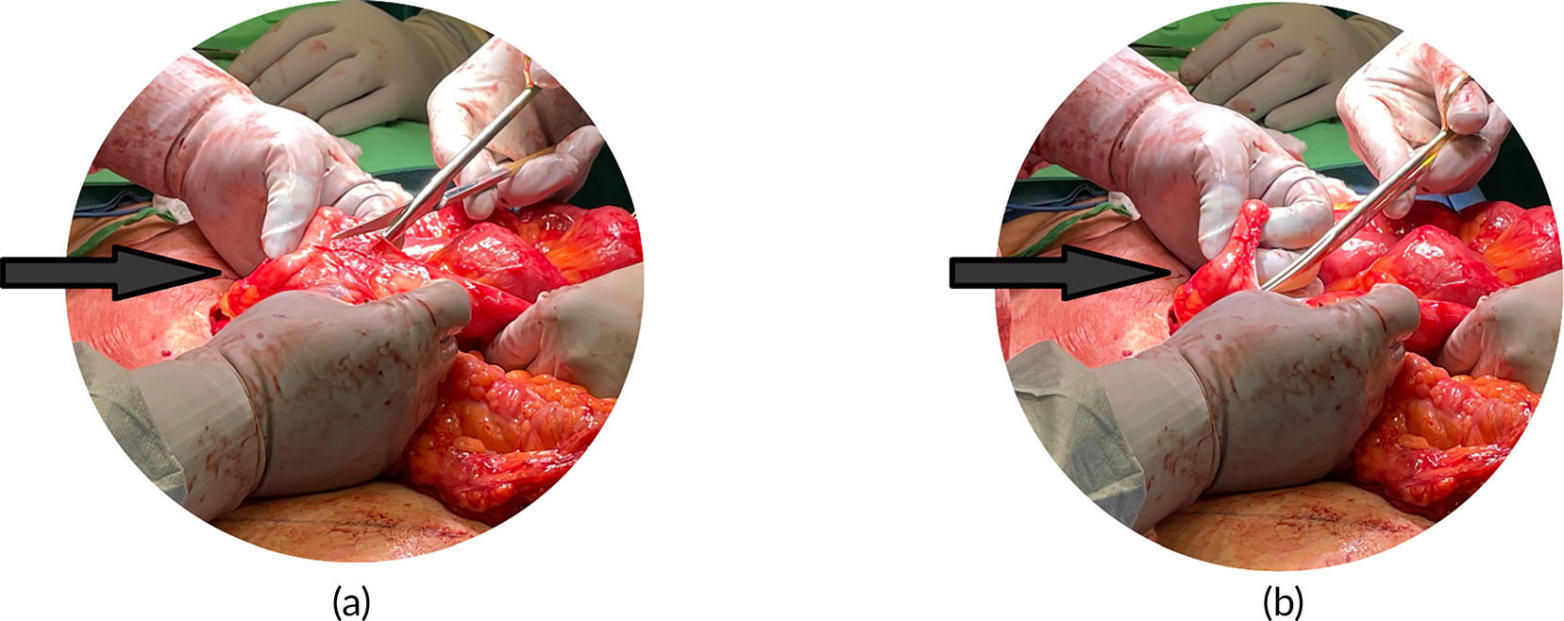
(a,b) Postsurgical adhesion lysis procedure during operation to treat ventral hernia recurrence (a. Before lysis procedure, b. After lysis procedure).

Postsurgical adhesions (Figure 1) are a serious complication for patients as they form fibrin scar tissue between the surfaces of organs and the internal wall of the abdominal cavity. Common abdominal surgeries such as appendectomies, caesarean sections, and intestinal anastomoses are followed with the inevitable consequence of postsurgical adhesion formation for 89%–95% of patients following one or more open abdominal operations.^1^, ^2^ Other abdominal surgeries involving incorporation of implanted medical material such as hernia repair with mesh implantation is associated with higher postsurgical adhesion formation.^3^, ^4^ The hernia repair with polypropylene mesh implantation have been found to contribute to adhesion formation to abdominal viscera, especially with small bowel.^5^, ^6^, ^7^ Common complications associated with postsurgical adhesions include small bowel obstruction, chronic pelvic pain, dyspareunia, infertility, and a higher risk of adhesion formation in subsequent surgical operations.^1^, ^8^, ^9^, ^10^, ^11^ Despite improvements in surgical technique, such as the development of minimally invasive surgery, which reduces tissue trauma and external contamination; adhesions still occur in up to 45%–64% of abdominal operations.^1^, ^2^, ^9^, ^12^

It has been postulated that postsurgical adhesions are triggered by tissue trauma or injury, and other contributory factors during the procedure, such as sharp, mechanical, or thermal irritation; ischemia; abrasion; and exposure to foreign bodies such as fiber, powder, irritating fluids, and airflow causing oxidative stress.^10^, ^13^ Additionally, it was observed in 1919 that healing of the peritoneum is different from external wound healing.^11^ Postsurgical adhesion formation has been found as a dynamic process triggered by traumatized tissues and resulting in the cascade effect of fibrin exudate, cytokine production, cell migration, vascular edema, and the suppression of fibrinolytic activity.^11^ This series of late cellular events includes the immune response and the fibrinolytic deposition forming a fibroblast‐rich scar tissue with collagen and extracellular matrix.^14^, ^15^ Whereas this late stage of adhesion formation is well understood, however, the dynamic and early events of postsurgical adhesion formation directly after surgery have not been well‐studied nor clearly understood. Thus, there are neither appropriate surgical procedures nor effective anti‐adhesion products currently available to prevent postsurgical adhesion formation.^15^, ^16^

By viewing adhesion tissue histologically, the composition of the internal scar tissue is known to contain a mixture of macrophages, eosinophils, red blood cells, tissue debris, mast cells, and fibroblasts.^11^, ^17^, ^18^ Milligan and Raftery et al. (1973) reported that postsurgical adhesions also include nerve fibers and endothelial cells which can play a role in angiogenesis and the formation and stabilization of any postsurgical adhesions.^19^, ^20^ Yanez‐Mo et al. (2003) suggested an alternative explanation for etiology of postsurgical adhesions from myofibroblast metaplasia.^21^ This concept has been supported by Sandoval et al. (2016), who determined that mesothelial cells undergo mesothelial‐to‐mesenchymal transition (MMT)^22^ to become myofibroblast cells and deposit extracellular matrix (ECM) with subperitoneal fibroblasts.^2^, ^11^, ^19^, ^23^ On the other hand, Tsai et al. (2018) recently observed via lineage tracing in a rat model that the early events of adhesion formation relied on activation of mesothelial cells rather than fibroblasts depositing matrix.^14^, ^15^, ^16^ Fischer et al. (2020) followed this previous study by creating a stressed mesothelial cell model to identify the cytoskeleton and calcium signaling channels that triggered the formation of postsurgical adhesion.^14^, ^15^

Based on our current understanding of the cellular mechanism, it has been identified that stressed mesothelial cells are a part of the earliest stages of the cascade towards triggering postsurgical adhesions. Current prevention methods that have been explored include physical barriers, gels, medications, as well as nanoparticles and gene therapy.^24^, ^25^ The most popular prevention devices approved by the Food and Drug Administration (FDA) have been Seprafilm®^26^ and Interceed**®**, which function only as a physical barrier to prevent adhesion formation.^27^ Since the introduction of Seprafilm® to the United States (US) market, multiple attempts have been made to petition the FDA for product recall due to concerns of complications associated with the inflammatory response of the implanted material such as peritonitis and severe foreign body reactions.^28^ Nevertheless, FDA has found no consistent evidence to support such complications and thus, Seprafilm® remains the current golden standard to reduce adhesion clinically. Based on the findings of three clinical trials and other relevant studies, Seprafilm® was found to be only 2%–8% efficient in preventing adhesions.^28^, ^29^, ^30^, ^31^ Although there are many different materials proposed for the prevention of postsurgical adhesions, the lack of understanding of the cellular mechanism of adhesion formation has made it difficult to develop an effective anti‐adhesion treatment.

Most of the current literature review of postsurgical adhesions involves only the surgical pathogenesis perspective or the selection of polymers and materials in the prevention of postsurgical adhesions. The goal of this literature review is to establish the current clinical perspective of adhesion formation, the postsurgical human peritoneum response, and the pathogenesis involved in adhesion formation. This will allow alternative clinical strategies to address the current issues of postsurgical adhesion formation associated with implantable biomedical devices. The perspective of the literature reviews provides a linkage between the integration of current clinical practice with biomedical engineering research.

### 1.1 Clinical significance of abdominal adhesion prevention

Postsurgical adhesion‐related issues place a large economic burden on the US healthcare system and negatively impact the standard of living of patients who undergo abdominal surgeries as well as complicating future abdominal procedures for surgeons.^2^, ^13^, ^32^, ^33^ There are significant hospitalization expenditures in the US that are more than $1 billion dollars per year for the patients who undergo abdominal and gynecological surgeries for treatment of adhesion‐related issues.^33^ Resulting from potentially prolonged hospitalization, patients are at an increased risk of internal organ injury, developing severe infections, and may require the need for physical rehabilitation before returning to normal daily functions.

Despite the economic impact of treating postsurgical adhesions, they are known to cause severe abdominal and pelvic pain,^34^ small bowel‐obstruction,^17^ complicate any secondary surgery,^20^ and infertility^35^ a for female patients. Although there are few studies that provide direct evidence to prove a correlation between chronic pelvic or abdominal pain and adhesions, nevertheless, some studies report that patients who experience adhesion lysis have less pain.^34^ Cheong et al. (2014) report that adhesions may cause infertility and pelvic pain by blocking the fallopian tubes and preventing oocyte retrieval.^18^ Ellis et al. (1999) reported that 64% of gynecological patients were readmitted within 10 years after surgery for adhesion‐related issues.^36^ Additionally, the abdominal procedures involving biomedical device implantation such as hernia or pelvic organ prolapse repair can significantly increase the possibility of forming postsurgical adhesions because of the foreign body response (FBR).^7^, ^35^, ^37^ The FBR is defined as a series of events initiated through the activity of foreign body giant cells that form scar tissue and/or a fibrous capsule.^37^ According to the previous study, it is not clear whether there is overlap between postsurgical adhesions and FBR, in that both involve an inflammatory response to injury or an implanted foreign material. It has been concluded that the presence of implanted foreign materials, such as a mesh or a suture, may play a role in the development of adhesion formation.^38^, ^39^ A study by Artsen et al. (2020) found that the presence of a mesh implant can exacerbate postsurgical adhesion formation by promoting a sustained foreign body reaction in the surrounding tissues.^40^, ^41^ They have noticed that this reaction involves the recruitment of inflammatory cells, which can secrete cytokines and other factors that promote fibrosis and adhesion formation.^38^, ^40^ Thus, the use of a mesh in abdominal procedures has been associated with severe complications, including the formation of postsurgical adhesions. In a study by Klinge et al. (2014), patients who underwent abdominal surgery with mesh implantation had a higher incidence of complications includes postsurgical adhesions, infections, or chronic inflammation based on the mesh explants samples collagen Type I and III ratio.^42^ Likewise, another study by Schreinemacher et al. (2013) found that patients who had mesh implanted with a layered coating develop less adhesion compared to mesh only.^4^ These findings suggest that the use of a mesh in abdominal procedures may increase the risk of adhesion formation and adding a layered coating have a potential to decrease the risk of adhesion formation.

It is important to be aware of the potential issues for adhesions following abdominal procedures, especially those involving mesh implantation, as they can lead to serious complications. At the same time, adhesion formation can also increase the risk of internal bleeding requiring blood transfusions, or visceral damage requiring intervention from other subspecialities or critical care. There are no effective surgical techniques, surgical barriers, or pharmaceutical products that have been successfully demonstrated to reduce the risk of clinical postsurgical adhesions. This is due to the lack of understanding of the physiological mechanism. Given the pressing need to address the formation of postsurgical adhesions, it is imperative to gain a comprehensive understanding of the underlying developmental process. Doing so will facilitate the application of current biomedical anti‐adhesion technologies, and pave the way for the design of novel, more effective therapeutic strategies.

### 1.2 Peritoneum anatomy

The peritoneum is part of the abdominal cavity, which is the largest cavity among the three serosal cavities found in the human body.^43^ The surface area of the peritoneum is equal to the skin, which is around 177 cm^2^/kg (body weight) for adults^17^ with a surface area of approximately 1.8 m^2^.^44^ The peritoneum serves to protect the abdominal cavity, minimize friction, facilitate free movement between abdominal viscera, resist or localize infection, and store fat, especially in the greater omentum.^17^, ^44^ The primary purpose of the peritoneal cavity is to provide a smooth surface layer over the abdominal viscera lined by a layer of mesothelial cells and a basal layer of connective tissue that is perfused with blood and lymphatic vessels to limit the mobility of viscera.^45^ It is also recognized as a dynamic cellular membrane with more complex structure and physiological functions and is known to participate in the embryogenesis of the primitive gut. The peritoneum has been shown to have control over fluid and cell transport; serving as a physiological barrier; participating in the induction, modulation, and inhibition of the immune system; generating tissue repair and the formation of scars; as well as preventing both adhesion and tumoral dissemination.^43^

**FIGURE 2:**
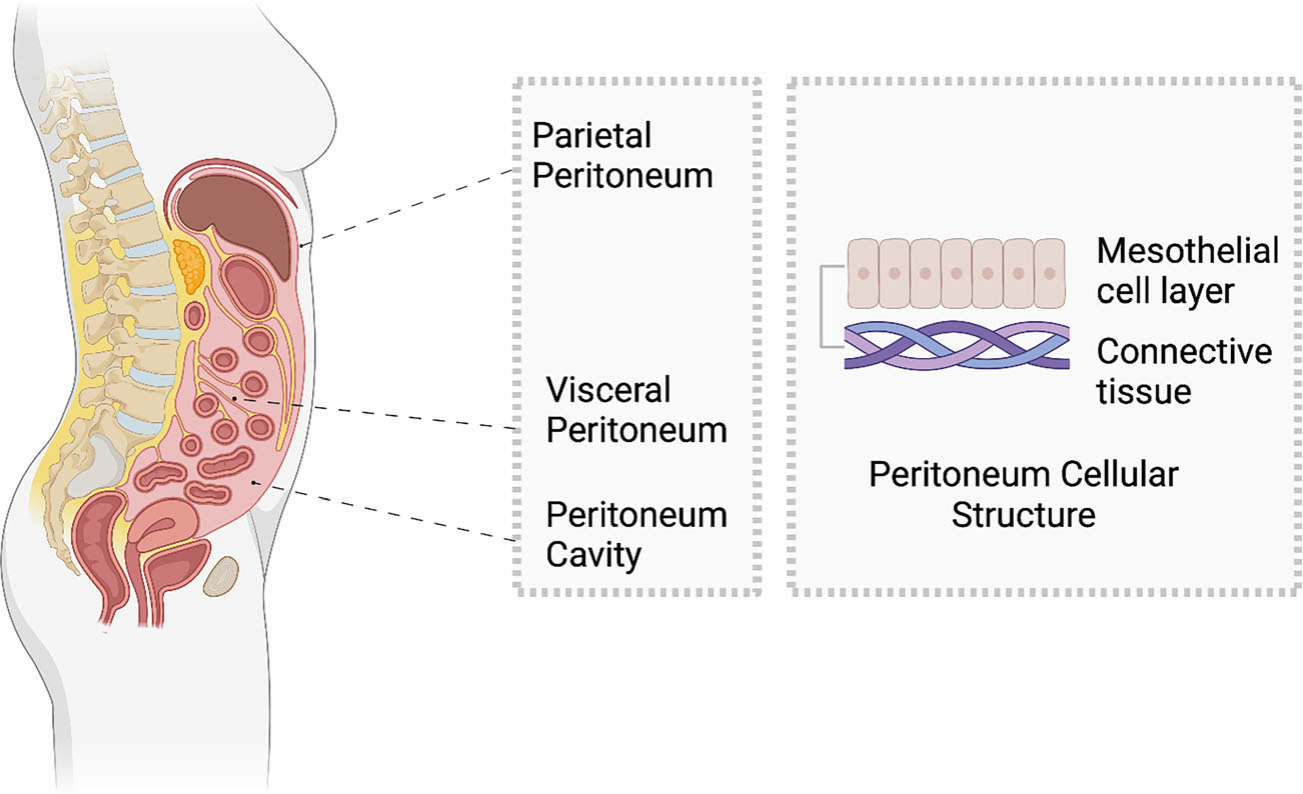
Anatomy and organization of the peritoneum including the visceral, parietal, and peritoneum cavities. The right‐hand image shows the cellular structure of two layers of mesothelial cells and connective tissue.

As shown in Figure 2, the inferior abdominal cavity is composed of the visceral peritoneum and the peritoneum cavity.^43^ The visceral peritoneum integrates with the outer serosal layers of organs, whereas the parietal peritoneum lines the inner surface of the abdominal wall as shown in Figure 2.^46^ There are numerous elastic fibers, especially in the deeper layers of the parietal peritoneum, and comparatively few connective tissue cells.^46^ The visceral layer of the mesothelial membranes covers the intra‐abdominal organs including liver, spleen, stomach, bowel, and the reproductive organs in females.^43^ Figure 2 also illustrates that the serosa is composed of two layers, namely loose connective submesothelial tissue and a mesothelial layer.^17^, ^43^ The mesothelial layer is a monolayer of cells that is attached to the basal lamina of connective tissue. Because they are loosely bound together to form an intracellular bridge or matrix, the peritoneum is susceptible to trauma or injury.^17^

The extracellular matrix provides a suitable microenvironment for tissue regeneration including different types of collagen (Type I and III), fibronectin, glycoproteins, fibroblasts, macrophages, as well as vascular and lymphatic vessels.^47^ The peritoneum forms a closed sac in the male and an open sac in the female because the ends of the fallopian tubes are not enclosed.^17^ An interesting phenomenon is that the anatomy of the female peritoneum is different around the ovaries. Based on the histology of the tissue around the ovaries, a layer of cells called the germinal epithelium covers the ovarian surface with a dense, irregular connective tissue layer called the tunica albuginea which is continuous with the peritoneum.^45^ Since the ovarian epithelium originates from the coelomic mesothelium, it therefore has similar structural, immunohistochemical and molecular characteristics as the peritoneal mesothelium.^47^, ^48^ The differences between female and male anatomy are due to the different types of tissue around the female ovaries. Referring to their histopathological observations, Beck et al. (1997) reported a higher frequency of postsurgical adhesions among female patients after abdominal/gynecological surgery compared to other types of operations in male patients.^49^ In other respects, the peritoneum lining the abdominal wall and the visceral organs are similar to males.^43^ Therefore, it is still not clear whether gender is a significant factor in developing adhesions.^50^

## 2 PATHOPHYSIOLOGY

Postsurgical adhesion can occur throughout the human body following various types of operations,^51^ such as cardiac,^52^ peritoneum,^1^, ^2^, ^8^ epidural,^53^ as well as tendon procedures.^54^ However, the pathophysiology of the different kinds of adhesion formation is still unclear as the tissue compositions are different between human cavities.^51^ The complications with abdominal adhesions affect patients' quality of life and make future operations more difficult are major factors in addressing postsurgical adhesions in the abdominal cavity. Currently, the most special component of in the formation of postsurgical abdominal adhesions are the origins of mesothelial cells^16^ and peritoneum large macrophages,^55^ which have been introduced to initiate the early events in adhesion formation.

As we introduced the internal abdominal cellular composition, the mesothelial cell is the most special component in forming the peritoneum, which is a single layer of mesothelial cells^56^ as it acts as a physiological barrier and participates in different biological activity. Therefore, the single layer of mesothelial cell function as a barrier or transportation medium by changing the apical‐basal polarity and intracellular junctions between the cells.^56^ Discovered complexes between the cells includes tight junction, adherens, and gap junction as well as desmosomes. Tight junctions and adherens provide structural support and semipermeable properties in regulating ions, water, or other soluble nutrients.^56^ However, the detailed pathway and interactions of these peritoneal components remain unknown. It has been found that the cell or fluids transport is known to regulate extracellular matrix (ECM) components through matrix metalloproteases (MMPs) and inhibitors (TIMPs) as well as generate procoagulant and fibrinolytic activity.^57^ Injured mesothelial cell also receive an influx of cytokines resulting in neutrophil and macrophage activation.^58^ In addition to the events involving postsurgical adhesions formation in the abdominal cavity, there are some similarities in other locations of the body that develop adhesions such as coagulation, inflammation, fibrinolysis, as well as ischemia.

Postsurgical adhesion formation is associated with the downregulation of both the fibrin deposition and fibrinolytic pathways during the wound‐healing process. When trauma or an incision occurs to the peritoneum, blood vessels increase their permeability and a release of histamines and cytokines initiate the inflammatory response.^11^, ^59^ The difference between normal and downregulated fibrinolytic activity depends on whether there is an ischemic condition.^59^ Under ischemic conditions, fibrinolytic activity is suppressed, and fibrin cannot be resorbed during the fibroblast proliferation process, which leads to fibroblast infiltration and scar tissue formation. Based on our current understanding, postsurgical adhesions are caused by the combination of wound healing and a series of cascades that includes, but is not limited to, hypoxia, coagulation, inflammation, and fibrin degradation.^60^ The cellular mechanism of these cascades is mainly dependent on the fibrinolytic system involving mesothelial cells.^8^, ^18^, ^60^ Neither the degree and details of each event, nor the whole cascade related to adhesion formation have been studied systematically. Different studies agree that after fibrin clot formation, immune cells and fibroblasts migrate to the clot and attach themselves starting the formation of a tissue scaffold. Angiogenesis also forms around the tissue leading to the deposition of fibrin and subsequent postsurgical adhesion formation.^47^, ^60^, ^61^ It has been known that the severity of acute inflammation is associated with adhesion formation.^16^ Tsai et al. (2018) demonstrated that the early events of adhesion formation involve the mechanical injury of mesothelial cells, which triggers the direct recruitment of immune cells including neutrophils and monocytes through chemokine release such as CXCL‐1 and MCP‐1.^58^ Following this mesothelial model, Fischer et al. (2020) identified a non‐specific calcium‐related signaling pathway, which is related to initiating the early events of mesothelial cells injury.^15^ Thus, mesothelial cells have been recently recognized as a clue to understanding early events of adhesion formation as well as associated inflammatory response.

### 2.1 Overview

**FIGURE 3:**
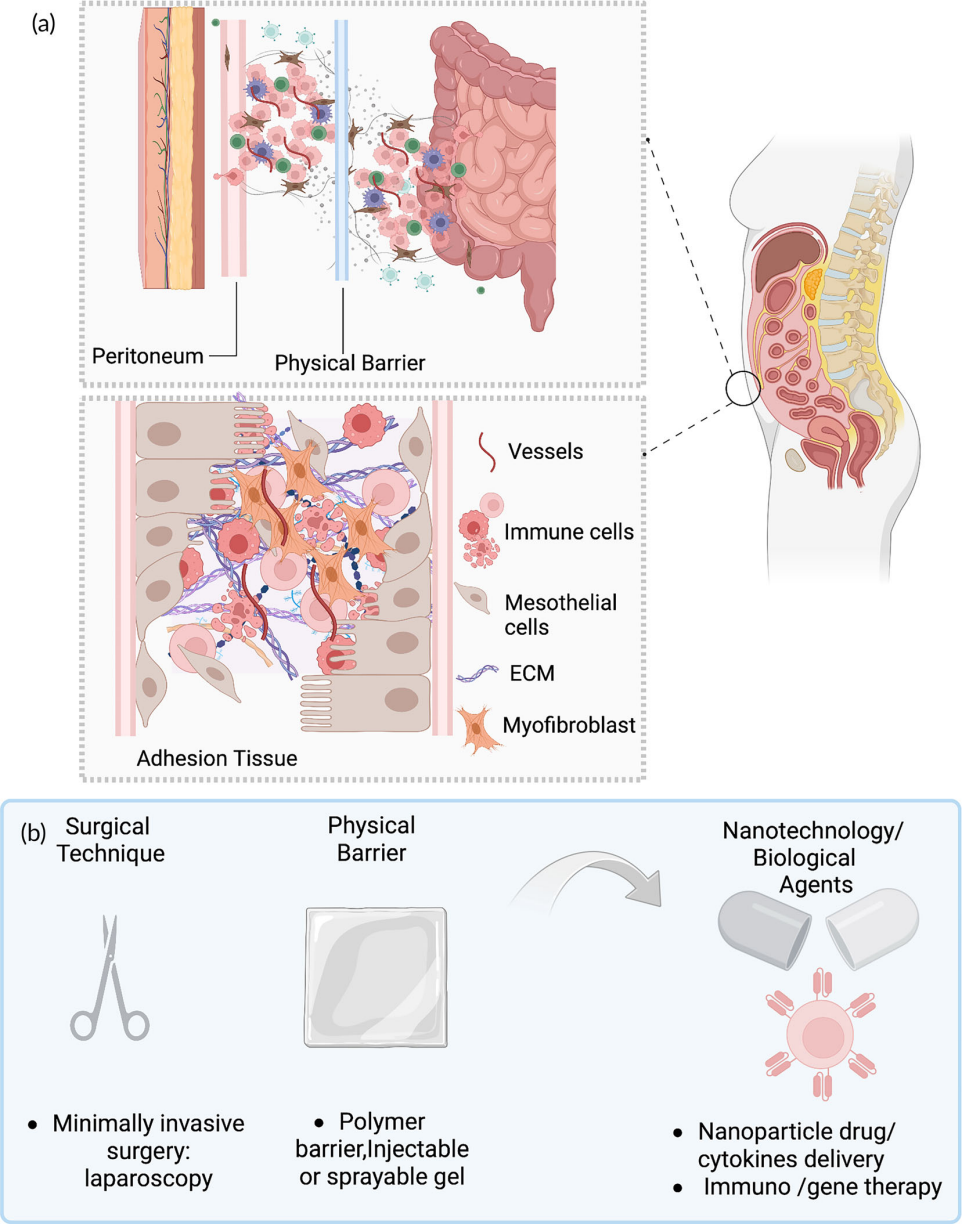
(a) A schematic overview of postsurgical adhesion composition and (b) prevention strategy of postsurgical adhesions.

When postsurgical adhesions are formed internally within the abdominal cavity, the series of events at the cellular level are elusive and difficult to understand and observe directly. From observations using a mouse model, postsurgical adhesions result from the downregulation of fibrin deposition and fibrinolytic activity, which is triggered by hypoxia, coagulation, inflammation, and fibrin degradation.^60^ The fibrinolytic system is the dependent factor in forming adhesions, which is supported by diZerega et al. (2000), who reported that the fibrin matrix generated during coagulation is associated with suppressed fibrinolysis.^11^ However, the traumatized tissue sites reduce blood flow and create ischemia that promote local fibrin matrix generation. diZerega et al. (2000) also reported that the development of intraperitoneal adhesions is a complex and dynamic process and leads to traumatized tissue binding together and forming a fibrin bridge.^11^ Over time, during the inflammatory response, the fibrin matrix is gradually replaced by vascular granulation tissue, which is characterized by the presence of macrophages, fibroblasts, and giant cells.^11^ These bridges are then organized by the elements of wound repair, which leads to the formation of a rich vascular network and the incorporation of neuronal elements. The mechanism of adhesion formation has been summarized by Diamond (2016), who claims that the release of histamine, cytokines, and growth factors triggers the coagulation cascade and inflammatory response, which leads to the release of serosanguineous fluid and the recruitment of immune cells from traumatized tissue.^59^ In addition, Sandoval et al. (2016) have reported that adhesion formation in humans consists of fibrin scar connective tissue, which is composed of extracellular matrix containing fibroblast‐like cells and capillaries.^22^ The cellularity and collagen matrix formed by fibroblast cells can vary depending on the number of cells. They also concluded that in most human samples, they were able to identify preserved mesothelial cells. In addition to fibroblasts, endothelial cells, and mesothelial cells, along with mononuclear inflammatory cells were also observed and characterized, including lymphocytes, mast cells, and macrophages, which were confirmed by immunohistochemistry markers such as CD45, tryptase, and CD68.^22^ Figure 3a provides a schematic illustration of the composition of adhesion scar tissue.

Given the complexity and magnitude of adhesion formation, this major healthcare concern has been underappreciated. The current stage of preventing or reducing adhesions includes two major strategies involving surgical techniques and the use of adjuvants as shown in Figure 3b.^62^ One improvement in surgical technique is the development of minimally invasive surgery or laparoscopic surgery, which reduces the extent of tissue trauma, improves hemostasis, and eliminates exposure to reactive oxygen species.^63^ However, this particular study^63^ concludes that the use of a laparoscopic technique does not reduce the frequency of adhesions compared to open surgery. This is because laparoscopic surgery is associated with longer operative times and high insufflation pressure, which can promote adhesion formation.^63^ In terms of using adjuvants, such as barrier agents or drugs, including anti‐inflammatory or fibrinolytic agents, they have been developed to prevent or reduce the risk of adhesions.^62^ While some of these adjuvants have shown promising results in animal models and clinical studies, there is no consensus on their efficacy and safety. Moreover, many adjuvants require expensive special expertise, and have side effects. The challenge of preventing or reducing adhesions is further compounded by the lack of good predictive animal models and the biochemical complexities of adhesiogenicity. Adhesions are a multifactorial process involving inflammation, angiogenesis, fibrosis, and matrix remodeling, which makes it difficult to develop targeted therapies. However, the potential method for preventing adhesions is still in development, and possible advanced bioengineering technology will direct future trends towards creating novel nanomaterials for drug delivery, developing, or modifying the biological components, such as utilizing nanoparticles or liposomes to deliver growth factors or cytokines and integrating nanotechnology with gene or immune therapy to regulate cellular events.

### 2.2 Physiological cellular mechanism

**FIGURE 4:**
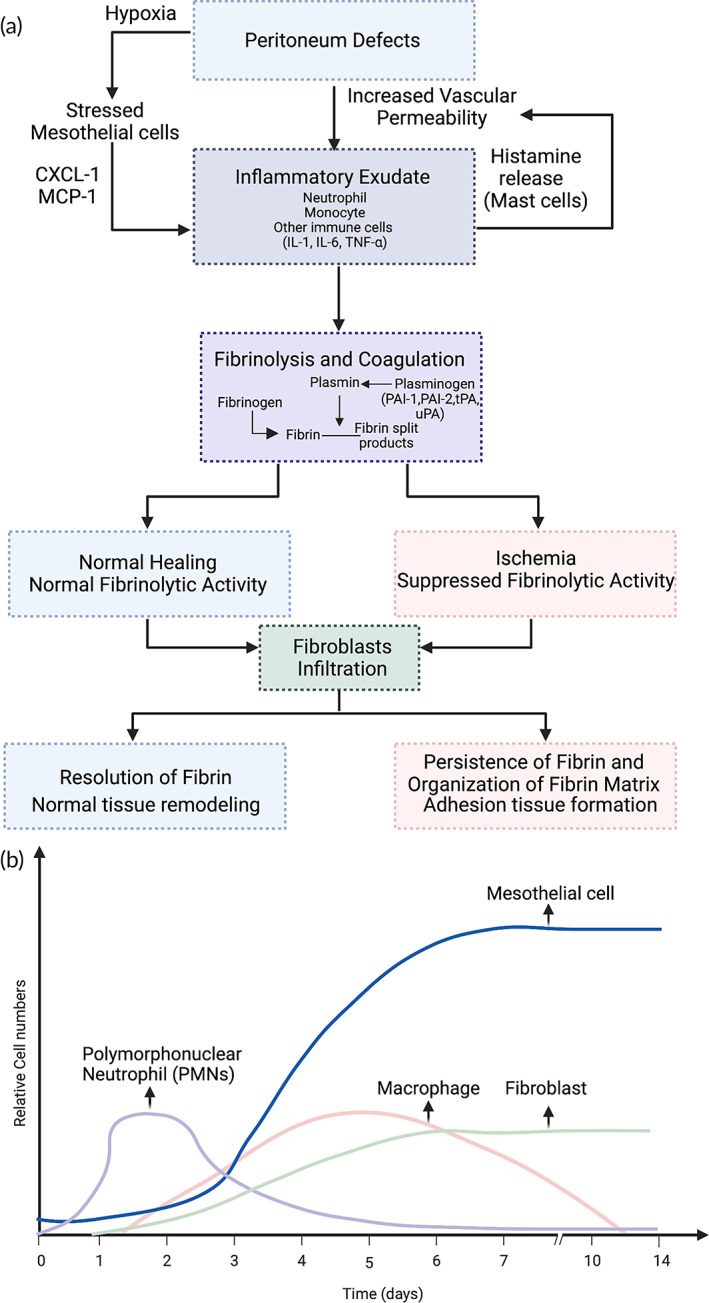
(a) Schematic illustration of the pathogenesis of adhesion formation showing the interactions between inflammation, ischemia or hypoxia, fibrinolysis, and coagulation. (b) Relative cellular numbers can change the rate of postoperative adhesion formation.

When trauma or a surgical incision causes an injury, it directly triggers a series of biological events or cascades which develops simultaneously in the peritoneum resulting in postsurgical adhesion formation.^11^, ^16^, ^18^, ^23^, ^60^ The schematic Figure 4a provides multiple events and pathways, which can potentially lead to postsurgical adhesion formation as well as significant changes in cellular composition during the healing process.^20^

Peritoneal trauma or injury begins with the disruption of the stomal mast cells, which release histamine and kinins while increasing vascular permeability.^64^ Platelet aggregation and plasma protein coagulation are essential components of the normal process of tissue repair. However, after surgical trauma or injury, abnormal regulation of fibrinolysis plays a significant role in adhesion development. The balance between fibrin deposition and fibrinolysis is determined by the level of plasmin.^65^ Plasminogen activator inhibitor‐1 (PAI‐1) is one of the main inhibitors of fibrinolysis, whereas plasminogen activators (Pas) activate plasminogen and mediate fibrinolysis activator and tissue‐type plasminogen activator (tPA).^66^ tPA provides a balance between pro‐coagulation and the fibrinolytic pathways, which are also regulated by PAI‐1.^66^ When abdominal injury occurs with an imbalance between tPA and PAI‐1, this can potentially lead to increased fibrin generation and the deposition of a fibrinous matrix which results in adhesion formation.^66^ Another important contributor related to the anticoagulation effect is antithrombin III which decreases thrombin activity.^65^ Fibrinolysis reduces the fibrinous matrix at surgical sites. However, even with fibroblast migration to the surgical site, the persistence of fibrin deposits leads to additional ECM formation and the potential to induce tissue adhesions.^67^ The increasing release of cytokines (IL‐1, IL‐6, and TNF‐α) from the inflammatory response could down‐regulate tPA activity, thereby decreasing the tPA/PAI ratio as well as the level of fibrinolytic activity.^68^ As a result, the fibrin‐rich matrix accumulates in the damaged serosa areas and persists longer than necessary, hence creating an environment that promotes the migration of macrophages. As these structures develop more capillaries and deposit more ECM, they are responsible for the formation of permanent adhesions within a week of undergoing surgery.^69^ The presence of a foreign body material, such as a mesh implant, or an infection will exacerbate this imbalance in fibrinolysis and result in excessive fibrin deposition and an increased risk of adhesion development.^67^

Ischemia or tissue hypoxia can trigger the release of free radicals, leading to changes in cellular activity such as increased cell proliferation, suppressed apoptosis, altered glucose metabolism, and changes in gene expression of various factors.^16^, ^70^, ^71^, ^72^, ^73^ During surgical procedures, the presences of free oxygen and nitrogen radicals can enhance DNA damage and increase the production of oxidized proteins.^64^

During normal wound healing, fibroblasts undergo apoptosis, associated with low oxygen tension and oxidative stress. Subsequently, more fibroblasts are attached to the wound site and change their phenotype to myofibroblasts and remodel the tissue. Imudia et al. (2005) observed that hypoxia induces cell apoptosis in normal fibroblasts, but less apoptosis in adherent fibroblasts.^74^ This is especially true when adherent fibroblast are exposed to hypoxia, because they can generate a higher degree of cell proliferation.^64^ Other researchers, such as Ara et al. studied hypoxia in a rat animal models and found that due to an increased generation of reactive oxygen species (ROS), a significant number of free radicals was produced during the first 5 minutes after hypoxia, followed by the appearance of antioxidant enzymes.^64^, ^75^ Other studies have mentioned that free radicals promote the expression of growth factors, including transforming growth factor beta (TGF‐ β), IL‐6, type‐1 collagen, and vascular endothelial growth factor (VEGF).^59^, ^76^ In addition, tissue hypoxia induces the production of superoxide, and fibroblasts exposed to superoxide could produce profibrogenic factors, such as TGF‐β and collagen I, which will eventually contribute to adhesion formation. With respect to peritoneal adhesions, fibroblasts change their phenotype to an adherent phenotype under hypoxic conditions which results in the increased production of VEGF to enhance the reoxygenation of hypoxic tissue that these clots represent.^74^

The adherent phenotype is characterized by an increase in the expression of fibronectin and type I and III collagen compared to normal fibroblasts, which are promoted by TGF‐β. The proteolysis of the deposited ECM is carried out by fibrinolytic and matrix metalloproteinase (MMP) systems, which are crucial in determine the fate of adhesions at this stage.^60^ Hypoxia resulting in oxidative stress, is believed to play an important role in the pathogenesis of adhesion formation.^16^, ^47^, ^64^ In addition, ensuring that the careful control of bleeding and the prevention of exposed tissue from drying is known to reduce the likelihood of adhesion formation.^77^

The study of cellular events by diZerega, G. S and Raftery enabled the cellular sequence of activities on the healing peritoneum defects in rats to be observed using scanning electron microscopy.^11^, ^19^ As discussed earlier, the important components of cellular change during adhesion development are shown in Figure 4b. At defects in the internal peritoneum changes in the cellular level change includes the appearance of polymorphonuclear leukocytes (PMN) among the fibrin strands 12 h after injury. That indicates that PMN's start to migrate from blood vessels to the wound site and the recruit macrophages as part of inflammatory response^11^, ^21^ between 24 and 36 h of the initial injury.^11^, ^19^ A single layer of macrophages covers the fibrin supported platelet plug at 48 h (Day 2). A few mesenchymal cells and the mesothelial cell islets interconnected by desmosomes and tight junctions have been observed at Day 2.^11^ At Day 3, while the macrophage is still the predominant cell type at the wound site, the surface also contains mesenchymal cells and a proliferation of fibroblasts in contact with each other.^11^ The mesenchymal cells and fibroblasts were in contact with each other, and the macrophage cells amount has decreased at Day 4.^11^ The earliest that the healing process appears to be complete is on Day 5 when a single layer of mesothelial cells has lined the surface.^11^ On Day 5–6, the number of macrophages decreases directly, and by Day 8–10, a continuous layer of mesothelial cells has created a continuous basement membrane.^11^

### 2.3 The role of the inflammatory response

An inflammatory response is activated by the peritoneum injury, in which the degree of acute inflammation appears to be associated with adhesion formation. Tsai et al. (2018) reported that an increased production of inflammatory mediators in the early inflammatory stage plays an important role in regulating ECM formation.^19^, ^58^ Additionally, activated platelets release various factors along with the fibrinogen and fibrin that are cleaved by thrombin and plasmin. These components act as chemoattractant for triggering the inflammatory response.^61^ Several studies have demonstrated the role of inflammation in preventing adhesion formation. In one of them, Hu et al. (2013) reported that the use of an anti‐inflammatory drug reduced the formation of peritendinous adhesion following surgery in a rat model.^78^ Adherent fibroblasts, associated with mRNA expression, were observed to increase their release of tumor necrosis factor‐alpha (TNF‐α) by 58% compared to normal fibroblasts, which increases the risk of surgical adhesions.^20^ Like TNF‐α, interleukin‐6 (IL‐6) is a pro‐inflammatory cytokine, that generates a systemic inflammatory reaction. Both TNF‐α and IL‐6 are known to regulate the coagulation cascade and the formation of fibrin.^20^ Recently, Uyama et al. (2019) have suggested that treatment with the IL‐6 receptor antibody reduces the risk of surgical adhesion formation.^79^ Other inflammatory mediators, such as IL‐17 and interferon gamma (IFN‐γ), are also believed to serve as potential therapeutic target molecules for the prevention of surgical adhesions.^20^, ^35^, ^36^ So, while anti‐inflammatory drugs as well as cytokines and small molecules have shown some efficiency in preventing adhesion formation, currently no specific target pathway has been identified, and no anti‐adhesion product developed that provides consistent results.

#### 2.3.1 Mesothelial cell injury

Since the major cellular component of the peritoneum layer is a loose monolayer of mesothelial cells, the mesothelial cells have been studied and recognized as the main character in abdominal tissue repair and the inflammatory process with the secretion of inflammatory cytokines, growth factors, and ECM components.^20^ Tsai et al. (2018) have reported that postsurgical adhesions originate from mesothelial cells. Podoplanin (PDPN) and mesothelin (MSLN) have been identified as the specific surface markers on the injured surfaces of mesothelial cells using a mouse model.^16^ Foster et al. also (2020) found that *JUN* expression was strongly induced and correlated with significant MSLN expression, and the inhibition of *JUN* has been found to minimizes adhesion formation.^80^ In another study, Fischer et al. (2020) reported that the calcium‐related signaling pathway of mesothelial cells initiates the formation of adhesions, suggesting that pathological changes of mesothelial cells act as the main component in the early adhesion cascade.^15^, ^16^ They also found that the calcium channel blocker drug is able to prevent in‐vitro changes to the mesothelial cell cytoskeleton as well as preventing adhesion in‐vivo model.^15^

A number of years ago, Yanez‐Mo et al. (2003) observed that under the influence of the transcriptional repressor SNAIL1, peritoneal mesothelial cells gradually lose their epithelial morphology and exhibit a decline in the expression of cytokeratin and E‐cadherin, as they undergo a transition from epithelial to mesenchymal cells.^21^ Most recently, Sandoval et al. (2016) confirmed that in the peritoneal cavity, mesothelial cells form a lining that undergoes a mesothelial‐to‐mesenchymal transition (MMT) in response to the pathological conditions. This process causes them to be transformed into myofibroblasts that are prevalent in peritoneal fibrotic tissue.^22^ In support of these findings, Uyama et al. (2019) observed that in response to TNF‐ α, adhesion‐associated myofibroblasts expressed mainly IL‐6 and INF‐ **γ** that originated from mesothelial cells induced adhesion.^79^

During adhesion formation, the release of vascular endothelial growth factor (VEGF) is an important event leading to neo‐angiogenesis.^81^ Previous studies have indicated that the mesothelial cells can secret significant amounts of VEGF during the MMT process.^82^ Animal experiments have shown that preventing the MMT can impede the development of peritoneal fibrosis and angiogenesis, which helps protect the peritoneal structure and function.^82^ In another unique study, Strippoli et al. (2020) demonstrated that caveolin1 and yes‐associate protein (YAP) drive mechanically induced MMT transitions. This demonstrates that there is cooperation between biomechanical and biochemical signals in the triggering of MMT transitions and represents a novel potential opportunity to intervene in mechanically induced disorders causing peritoneal fibrosis, such as postsurgical adhesion development.^83^

Injury to mesothelial cells is associated with the secretion of different cytokines, chemokines, and growth factors and the migration of immune cells to the site of injury, thereby initiating an acute inflammatory response.^84^ Mesothelial cells produce a multitude of cytokines and growth factors which can regulate the inflammatory responses and tissue repair. The stimulation of mesothelial cells induces the pro‐inflammatory cytokines and chemokines to release IL‐1, IL‐8, monocyte chemoattractant protein‐1 (MCP‐1), and IFN‐ γ, all of which are associated with adhesion formation.

#### 2.3.2 Macrophage recognition

The injured peritoneum has been observed through live intravital microscopy. By imaging living cells while they are in a complex multicellular environment, it has been possible to characterize the sequence of the cell recruitment.^85^ A surprisingly finding by Zindel et al. (2021) is that monocytes and macrophage migrates to the injury sites and cover the lesion within 15 min of injury.^55^ This finding is consistent with the previous study that showed macrophages are the most prevalent cell type within the injured peritoneum on Day 1 after adhesion induction.^86^ Thus, the recruitment of monocytes followed by migration of macrophage was significantly faster than the recruitment of neutrophils.^55^ So, this new finding changes the current understanding of initial adhesion formation cascades as it was previously believed that neutrophils played a significant role. There is a specific type of macrophage called large peritoneal macrophages (LPM) that is characterized by the expression of CD102 (Icam2) with transcription factor GATA6.^85^ It undertakes the role in cleaning bacteria by phagocytosis and also participating in the adhesion formation process.^55^ The observation of macrophages has recognized the importance of the passive transportation of the peritoneum fluids as it allows macrophages to aggregate rapidly after the injury and rapidly respond to Damage‐associated molecular patterns (DAMPs).^55^ The cell–cell aggregates usually take place surrounded by blood vessel wall.^85^ Peritoneal macrophages models have been proposed to be activated by DAMPs to Pattern recognition receptors (PRR). This activation can lead to the polarization of macrophages into an inflammatory M1 phenotype.^85^ M1 macrophages play an important part in the degradation of the ECM during inflammatory response.^87^ M1 macrophages produce pro‐inflammatory cytokines such as TNF and IL‐1, as well as reactive oxygen species and other mediators that promote inflammation and tissue damage. Rajab et al. (1997) suggested that enhanced peritoneal macrophages by protease peptone increased the activity of plasminogen and inhibited the development of adhesion formation.^88^ Additionally, macrophage depletion and irritation/injury of the peritoneum resulted in peritoneal adhesion formation replicated in a mouse model.^89^ A previous study suggested that prior depletion of macrophages could induce a significant neutrophil influx into the tissue in response to lipopolysaccharide.^87^ Macrophages aggregation have participated in peritoneal wound healing process as well as cause inflammation. The role of macrophage aggregation is not clear whether it cause inflammatory or anti‐inflammatory.^85^ The above findings suggest an important role of macrophages in adhesion phenotype, but their differentiation is still unknown. Macrophage function depends mainly on differentiation status, and its differentiation could affect wound healing in several organs.^90^ A recent study demonstrated that although activated mesothelial cells induced the recruitment of monocytes through monocyte chemoattractant protein (MCP), the number of accumulated macrophages was markedly decreased throughout the adhesion formation process.^58^ Macrophages could produce plasminogen activator inhibitors and tissue plasminogen activators that modulate fibrinolysis and inflammatory response.^91^ Macrophage disappearance reaction (MDR) have been proposed by Nelson and Boyden, not as a specific reaction, but a response correlated to the increased inflammatory cytokines in the peritoneal fluids as well as the influx of pro‐inflammatory leukocytes.^85^ However, there are still largely unknown changes in macrophage cellular events. Therefore, understanding of the functions and mechanisms of the various macrophage subpopulations can help to open new strategies for the prevention of surgical adhesion formation.

## 3 POSTSURGICAL ADHESION PREVENTION STRATEGIES

Despite significant efforts to understand the mechanism of postsurgical adhesion formation,

there is still insufficient evidence to fully explain all the cellular and molecular pathways involved in the formation of adhesions. Various strategies have been proposed to address this issue. Nevertheless, postsurgical adhesions continue to be reported. Even after adhesiolysis to remove the bands of scar tissue between adjoining organs and peritoneal structures, adhesion development still occurs in 80% of patients regardless of the type of procedure.^59^ Thus, postsurgical adhesions continue to be an intractable issue for all surgeons, not only because there are currently no effective clinical procedures or products available to prevent postsurgical adhesion formation, but also because there are two main factors that need to be addressed. The first factor is the lack of understanding of the pathological mechanism of adhesion formation. The second factor is the absence of a reliable and reproducible in‐vitro model that can be used to study and evaluate the mechanism of postsurgical adhesion formation. Most potential anti‐adhesion candidates can only rely on in‐vivo models to evaluate the efficiency in preventing postsurgical adhesion, which brings in the question the accuracy and consistency of the experimental results.

In this chapter, we review and summarize the prevention strategies and for postsurgical adhesions. We discussed the current research on various forms of polymers, pharmaceutical product, and nanoparticles, as well as the potential use of gene therapy to prevent the formation of postsurgical adhesions.

### 3.1 Commercialized and FDA‐approved anti‐adhesion products

The application of a physical barrier is the most common and low‐cost method used in preventing postsurgical adhesions. In general, the traditional anti‐adhesion membranes work as a separating layer to avoid contact between surgical tissue sites and other organs.^59^, ^92^ They act as a boundary to inhibit the formation of tissue fibrin bridges during the tissue regeneration stage, hence reducing postsurgical adhesion formation.^28^, ^93^ Currently, there are three common forms of commercial products used to treat postsurgical adhesion; which include gel, films, and solutions.^92^ Most of them rely on a biocompatible material that has the potential to prevent adhesion formation. For example, hyaluronic Acid (HA), oxidized regenerated cellulose (ORC), polylactic acid (PLA),and polyethylene glycol (PEG) are all possible candidates. However, none of them have been consistently proven to possess high efficacy for preventing adhesions. Additionally, there is a lack of studies about the long‐term effects and efficacy of the implanted anti‐barrier cytotoxicity or side effect profiles. The current data from clinical trials points to the efficiency rates of those products ranging from 26% to 70% in clinical studies.^16^, ^78^, ^85^, ^86^, ^87^, ^88^ Some other products such as Interceed® (Ethicon, Somerville, NJ) and Adept® (Ethicon, Somerville, NJ), have been approved by the FDA for gynecological surgery only.

To better understand how these materials play a role in preventing adhesions, numerous animal and human trials have been performed. Conflicting results from these studies have only raised further questions of the true efficacy of these materials. A major disadvantage of using a barrier method is the need for fixation to a particular anatomical site. The current clinical gold standard is Seprafilm®, which is composed of carboxymethyl cellulose (CMC) and HA. Even with Seprafilm®, the clinical significance of this barrier product is questionable in light of several petitions to the FDA for its recall. As a result, commercial barriers still have a long way to go before they can be used with confidence to resolve this problem or integrated new biotechnology into creating the next generation anti‐adhesion barriers.

Table 1 lists the commercialized products on the US and international markets, and describes their materials, product form, approval time, efficacy (according to clinical trials) and their claimed function and performance. Typically, the anti‐adhesion barrier should follow the principles listed as follows^27^:

1. Effectively separate tissues.
2. Has a long half‐life of at least 7 days after surgery within the peritoneal cavity.
3. Absorbed or metabolized without initiating a marked proinflammatory response.
4. Remain active and effective even when exposed to blood.
5. Does not compromise wound healing.
6. Does not promote infection.

**TABLE 1:** Commercialized anti‐adhesion products

Brand (company)	Material	Format	Approval	Intended use	Efficacy	Claimed property	Disadvantage
Coseal® (Baxter Healthcare Inc, Deerfield, IL)	Polyethylene glycol (PEG)	Hydrogel/sealant	CE‐2000 FDA‐2003	Hemostasis product; cardiac surgeries	No toxicity^94^	Biodegradability; hemostasis^95^	Swelling may induce compressing; curing time needed
Repel‐CV® (SyntheMed Inc, Iselin, NJ)	Polylactic acid‐Polyethylene glycol (PLA‐PEG)	Film	FDA‐2009	Pediatric cardiac surgery	26%^96^	Low cell‐adhesion; flexible; biodegradability	Fixation difficulty; efficiency same as seprafilm
Hyalobarrier® (Nordic Pharma, Paris, France)	Hyaluronic acid (HA)	Hydrogel	CE‐1999 not approved in US	Abdomin‐pelvic area	17.75%^97^	Biocompatibility; bioresorbability	Lower efficacy comparing with other products^98^
SprayShield® (Coviden; Dublin, Irland)	Polyethylene glycol (PEG)	Spray	CE‐2008 not seeking FDA	Surgical applications	30%^89^	Bioresorbability hydrogel form onsite	Complications reported in few studies^98^
Adept® (Ethicon, Somerville, NJ)	Icodextrin (4%)	Solution	CE‐1999 FDA‐2006	Gynecological surgery	30%^99^	Delayed bioresorbability	Anastomotic wound healing problems^100^
Interceed® (Ethicon, Somerville, NJ)	Oxidized regenerated cellulose (ORC)	Film	FDA‐1989	Gynecological surgery	70%^86^, ^101^	Low inflammatory response; mufcoadhesive; delayed bioresorbability	Fixation difficulty; hard to handle; efficiency rate is debating
Seprafilm (Baxter, Deerfield, IL)	Carboxymethyl cellulose (CMC)/hyaluronic acid (HA)	Film	FDA‐1996‐2022	General surgery	33%^102^	Low inflammatory response; muco‐adhesive; bioresorbability	Fixation; low efficiency for preventing adhesion

Besides the FDA‐approved barrier products, there are still a lot of studies working on or attempting to improve the properties of biomaterials to prevent adhesions. Liao et al. (2023) summarized the studies for fabricating physical anti‐adhesion membrane by manipulating the surface of the biomaterials and exploring the combination of multifunction's biomaterials in order to reduce postsurgical adhesions.^51^ Erdi et al. (2023) also take the advantages of current nanotechnology and introduced the most recent development of polymer types nanomaterials to utilize them as a more effective adjuvant surgical tool for surgical treatment, which has opened a new window in combining functional biomaterials to current clinical settings.^103^

### 3.2 Cellular Modulation

Many studies have researched the use of biomaterials as a barrier‐type anti‐adhesion membrane for clinical practice. To date, the reported clinical efficacy of these biomaterials has been disappointing. Despite this, the inert property of barrier materials has a significant untapped potential to be a component in the integration of drugs and/or biological agents to improve the efficacy of preventing adhesions and further expand their clinical use. Thus, there are multiple studies currently being performed on assess the incorporation of different types of drugs targeting anti‐inflammatory, anticoagulative, and fibrinolytic properties, as well as growth factors in conjunction the biomaterials. However, the duration of these studies is limited to the short‐term effects of drug delivery. Further research needs to be performed in assessing long‐term mechanisms and clinical efficacy during the wound‐healing process.

Nanoparticles have been suggested as an alternative controlled drug delivery system because of their unique physical properties, which include uniform, modifiable particles, and various pore sizes allowing for a better control of the duration of drug delivery. Additionally, nanoparticles have other highly desirable properties including high surface area, large pore volume, and superior biocompatibility.^104^ The use of biologically active substances loaded into nanoparticles offers an attractive opportunity to deliver anti‐adhesion therapeutics in a targeted and personalized manner. This approach may address and resolve the side effect of many agents. Additionally, nanoparticles can be incorporated into polymeric fibers and membranes, which would allow them to be used with current barrier methods. Table 2 lists the recent research that are utilizes nanoparticles and present their performance in both in‐vitro and in‐vivo models.

**TABLE 2:** Anti‐Adhesion Research

Function	Nano‐scale carrier	Drug/gene?	Membrane/solution	In‐vitro result	In‐vivo result	References
Fibroblasts proliferation; infection	Ag nanoparticles	N/A	Electrospun poly(l‐lactide) PLLA membrane	Less fibroblasts proliferation (Not related to adhesion pathology, can only prove no cell attachment for antibacterial property)	N/A (lack of evidence for proving anti‐adhesion property)	105
Fibroblasts attachment; infection; inflammatory response	Ag nanoparticles	Ibuprofen	Poly (ethylene glycol) (PEG)/Poly (caprolactone) (PCL) Shell Hyaluronic acid (HA) Core	Less cell attachment and good biocompatibility (not related to adhesion pathology, can only prove no cell attachment for antibacterial property).	Less inflammation and adhesion have been observed on core‐shell nanofibrous membranes directly as well as through histology (no inducing adhesion model and adhesion score to quantify the data).	106
Collagen Type I expression	Dextran glassy nanoparticles (DGNs)	bFGF	Electrospun PLLA membrane	Less multipotent C3H/10 T1/2 cell attachment with good biocompatibility	A blunt dissection for separating the tendons and peritendinous tissues for treated group compared to control with sharp dissection as well as quantifying the breaking force for all groups.	107
Collagen Type I and PCNA protein expression	PLGA Nanoparticles	Constructed an overexpressed bFGF+VEGFA genes with GFP in pEGFP‐N1 plasmid	PEI Polymer suspension	Low cytotoxicity on samples carried out with MTT cell viability test, and high transfection rate of the tenocytes cell culture determined the effectiveness of the gene delivery.	Adhesion score of white Leghorn chicken in‐vivo model significantly decreased with treatment and the tendon ultimate strength increased representing good healing results.	108
TGF‐β1	Polylactic‐co‐glycolic acid (PLGA) Nanoparticles	miRNA based RNA interference (RNAi) plasmid	Polyethyleneimine (PEI) polymer suspension	Low cytotoxicity on samples carried out with MTT cell viability test.	Adhesion score, real‐time PCR, western blot of chicken tendon repair model with nanosphere/plasmid effectively inhibit the expression of TGF‐β1 for over 70% till week 6.	109
Phosphorylation of ERK1/2 and SMAD2/3	N/A	Fresh amnions (include TGF‐β1, bFGF, VEGF, and PDGF)	PCL electrospun nanofibres	Higher cell viability and proliferation on tenocytes and fibroblasts showed a better proliferation activity compared to control, 7 days cell culture also showed ERK1/2 and SMAD2/3 protein expression higher than control.	Adhesion score macroscopic and microscopic decreased on PCL electrospun treated group compared to control and amnions group. Histological healing percent also showed the best on PCL electrospun group.	110
Down‐regulate proinflammatory gene and protein expression (Tgfb3 and Tgfbr2), up‐regulate inhibitory proteins Smad6 and Smad7, ghrelin induce blockage of TGF‐β signaling	N/A	Ghrelin	N/A	N/A	In‐vivo mice model showed that ghrelin inhibits the TGF‐β/Smads and p‐38 MAPK signaling pathways activation during the inflammatory response at the onset of injury before the granulation‐remodeling phase occurs, which significantly reduce the local inflammatory mediators and therefore prevent adhesion	111
Enhance fibrinolytic and enhance EGF‐HER1 signaling by regulating macrophage	N/A	Plasminogen activator inhibitor 1	Carboxymethylcellulose (CMC)	N/A	The results have showed that the incidence of adhesions have been reduced but not eliminated in gauze induced adhesion rat model. The pharmacologic inhibition of PAI‐1 can prevent adhesion without the fear of bleeding, anastomotic disruption, and wound dehiscence.	112
PAI‐1 and tPA levels	N/A	tissue‐type plasminogen activator (tPA)	Poly(ε‐caprolactone)‐poly(ethylene glycol)‐poly(ε‐caprolactone) (PCEC)	N/A (Only drug release efficiency carried out in‐vitro experiment for a 14‐days slow release)	Adhesion score and frequency showed lower score for tPA‐hydrogel treated group, H&E staining as well as tPA and PAI‐1 level carried out with tPA‐hydrogel system	113
Fibrin deposition; angiogensis; macrophage infiltration	Peritoneal cell sheet (Include mesothelial cells and fibroblasts)	N/A	N/A	N/A	Peritoneal cell sheet transplantation prevents tissue adhesion, fibrin deposition, angiogenesis, lymphomagenesis, and macrophage infiltration in a rat cecal cauterization adhesion model.	114
Angiogenesis inhibitor, COX‐2 inhibitors drugs	N/A	Celecoxib, rofecoxib	Methylcellulose, silicone patch	N/A	Rats treated with selective and nonselective COX‐2 inhibitors had significantly fewer adhesions than control animals. Adhesions from mice treated with celecoxib had reduced microvessel density compared with rofecoxib, the nonselective COX inhibitors, and control animals.	205^115^
Promote mesothelial cell proliferation; enhance fibrinolytic activity	N/A	Keratinocyte growth factor (KGF)	Sodium hyaluronate (HA) gel	N/A	The combination of KGF and HA can inhibit the severity of the fibrous changes in the adhesion induced rat model	116
Mesothelial cells Actin cytoskeleton (calcium channel); Anti‐adhesion	N/A	Small molecular calcium channel blocker (Bepridil, CK‐666, Rhosin, and Golgicide)	2% Cellulose	Unstressed cytoskeleton effect of GFP labeled mesothelial cells in situ induced in‐vitro assay mesothelial cells with calcium channel blocker drug treatment.	Adhesion score of rats with different dosage of the calcium channel blocker treated and tested 27 targets of signaling pathway with single‐cell transcriptomics analyses to prove the adhesion related gene expression.	15
T cells in TLR4/MyD88/NF‐κB signaling pathway	Polylactic acid (PLA) nanoparticles	ligustrazine	Potassium dihydrogen phosphate/ sodium hydroxide solution suspension	N/A (Only drug release efficiency carried out in‐vitro experiment)	Adhesion score, Masson trichrome (MT) staining, ELISA, western blotting, Immunohistochemistry and PCR shows significantly lower adhesion formation due to inactivating the TLR4/MyD88/NF‐κB pathway as well as regulating the downstream cytokine expression.	117
Anti‐adhesion, immune cell infiltration	Mesoporous silica nanoparticles	Ibuprofen	Electrospun PLLA composite	N/A (Only drug release efficiency carried out in‐vitro experiment)	Adhesion score and H&E staining showed an improvement for anti‐adhesion and anti‐inflammatory results of treated group with drug‐loaded nanoparticles compared to control.	78
Anti‐adhesion	PLGA Nanoparticles	N/A	Aldehyde‐ and hydrazide‐modified hyaluronic acids (HA)	Low cytotoxicity on murine mesothelial cells	Adhesion score of rats and rabbits model showed no adhesion on over 60% of animals treated with this gel system compared to over 50% of animals on control group with severe adhesions.	118
Anti‐adhesion, reduce formation of intrauterine adhesion	N/A	N/A	Crosslinked hyaluronan gel	N/A	60 patients were divided into two groups: Group 1 received curettage plus crosslinked hyaluronan gel (intervention group), and Group 2 received curettage alone (control group), Results showed hyaluronan gel reduce the formation of intrauterine adhesions in women after curettage for retained placental tissue after medically induced or spontaneous pregnancy loss in the second trimester	119
Anticoagulation	N/A	Heparin	Carboxymethylcellulose (CMC)	N/A	Adhesion score have significantly decreased with tiny doses of heparin, in combination with CMC 4% gel in two different animal models. (Two different types of animal models: Avascular knot model and Cecal abrasion model)	120
Antibacterial, hemostatic, anti‐inflammatory and biocompatibility	N/A	N/A	Nanochitosan membrane barrier	Higher adherence and biocompatibility (over 90%) on the material with a mouse fibroblasts L929 cell culture.	Rats' open abdominal cavity model proved nanochitosan membrane can reduce the invasion of inflammatory cells into the wound and inhibits the synthesis of collagen to prevent postoperative adhesions	121
Intracellular glutathione levels; Anti‐inflammatory; antioxidant	N‐acetylcysteine (NAC) nanoparticles	N/A	N/A	N/A	Adhesion score and C‐Reactive protein concentration showed a better result of lower doses of Nano‐NAC (50 and 75 mg/kg) compared to higher dose and control.	122
Anti‐inflammatory and anti‐oxidant; anti‐proliferation of fibroblast	N/A	Phlorotannin	Poly (ε‐caprolactone) film	Less fibroblasts proliferation and inhibit nitric oxide production (Not related to adhesion pathology, can only prove no cell attachment for antibacterial property)	N/A	123
Anti‐inflammatory, antioxidant, and antifibrotic effects	N/A	Pirfenidone	Carboxymethylcellulose (CMC)	N/A	Rat model revealed that pirfenidone has remarkable protective effects against postoperative intraabdominal adhesions and inflammation with a decreased mRNA expression levels and increased MMP‐9 concentration detected in peritoneal tissue.	124
Anti‐inflammatory	N/A	N/A	Human amniotic membrane	N/A	There were significantly fewer adhesions in the amniotic membrane group (2) versus hyaluronic acid (3) group (*p* = 0.01).	125
Anti‐inflammation and anti‐oxidation effects	N/A	20(S)‐ginsenoside Rg3	20(S)‐ginsenoside Rg3‐loaded methoxy poly (ethylene glycol)‐block‐poly(L‐lactide‐co‐glycolide) (mPEG‐b‐PLGA) electrospun membrane (PEM/Rg3)	N/A	PEM/Rg3 membranes showed a significantly reduce of the peritoneal adhesion in rats model.	126
Anti‐inflammatory, promote healing, reduce fibrous adhesion	Nanomicelles	Lipophilic gold nanorods and curcumin	Lipophilic gold nanorods stabilized with the surfactant agent hexadecyltrimethylammonium bromide	N/A	The rat model showed controlled release of curcumin from these nanomicelles can effectively reduce adhesion formation while promoting tendon healing	127
Anti‐inflammatory; antioxidant; RhoA/ROCK signaling pathway	N/A	Rho GTPases	Sodium aescinate (AESS)	The cytotoxicity assay was used to decide the concentration of the AESS with cell proliferation inhibition condition.	AESS significantly reduces postoperative adhesion formation in a rat model; AESS increases the secretion and activity of tPA in peritoneal fluid and decreases FIB levels in plasma. AESS‐treated groups showed that the secretion, activity, and expression of tPA in rat peritoneum were notably increased.	128
Physical barrier for pericardial adhesion	PLA–PEG nanoparticles	N/A	HPMC‐C12	N/A	Adhesion score of rats and sheep model treated with a PNP hydrogel adhesion barrier exhibited formulation‐dependent results, solid‐like PNP hydrogel formulations (G′ > G′′) 1:5, 1:10 and 2:10 all formed physical adhesion barriers that significantly reduced the incidence and severity of adhesions when compared with the untreated control group.	129

According to the list, polymeric nanoparticles prepared from polylactic acid (PLA) and polyethylene glycol (PEG) are among the more popular options because of their ease of preparation and their FDA‐approved biocompatibility. Some studies claim that due to their unique nano‐scale morphology, the attachment of cells is significantly reduced.^129^ Silver nanoparticles are another commonly used biomaterial for this purpose. The silver (Ag+) ion can reduce the incidence of infection due to its anti‐bacterial effect.^105^ However, its cytotoxicity may raise concerns over long‐term use. Another newly emerging carrier involves the use of cell or cell‐derived products, which may further promote the biocompatibility and a more bio‐active content that can effectively regulate the cell responses.^110^

There is wide range of different types of drugs that can be incorporated within these nanoparticles, and most of them are targeted to either modify the inflammatory response or to reduce the formation of fibrosis tissues. One of the common drugs, such as Ibuprofen is a candidate that works on the anti‐inflammatory process and result in less cell adhesion. This suggests that by blocking certain pathways one can prevent postsurgical adhesions from occurring.^78^, ^106^ Thus, instead of relying on a pro‐formulated medications strategy, the involvement of substances that cause cellular and/or cellular modulation effects may be able to better control these identified pathways more effectively. For example, Zhou et al. (2015) used TGF‐β1 mi‐RNA plasmids in a chicken tendon adhesion model to target and downregulate TGF‐β1. This study showed that using a plasmid‐nanoparticle as a non‐viral vector for gene therapy can potentially be beneficial in adhesion prevention.^109^ Bianchi et al. (2016) applied ghrelin that can down‐regulate the proinflammatory gene and protein expression (TGF‐β3 and TGF‐β2), up‐regulate the inhibitory proteins Smad6 and Smad7, and induce blockage of TGF‐β signaling to reduce the local inflammatory response.^111^ Growth factor is also used frequently in reducing adhesion formation, by promoting the growth of the normal cells in healing processes.^108^, ^112^, ^116^

The in‐vivo animal model is currently the most sufficient and reliable method to test and evaluate anti‐adhesion candidates. Various animal models have been used in performing in‐vivo studies, however, the most common in‐vivo animal peritoneal model is the rat model. Additionally, there have been multiple methods introduced in previous studies to standardize adhesion inducing models, such as using visceral and parietal brushing, mechanical denuding and sutures, creating ischemia buttons as well as using electrocoagulation, etc.^130^

The prevention of postsurgical adhesions through clinical approaches is currently limited to those biomedical devices that have been approved by the FDA. Only physical barrier types of commercial products have been applied clinically, leaving significant opportunities for further improvement. At the present time various studies that are being conducted that aim to enhance the efficacy of preventing adhesions through alternative pharmacological delivery methods. These approaches have the potential to achieve higher efficiency rates and potentially yield promising results for preventing adhesions in future clinical practice.

### 3.3 Future Therapeutic Strategy

**FIGURE 5:**
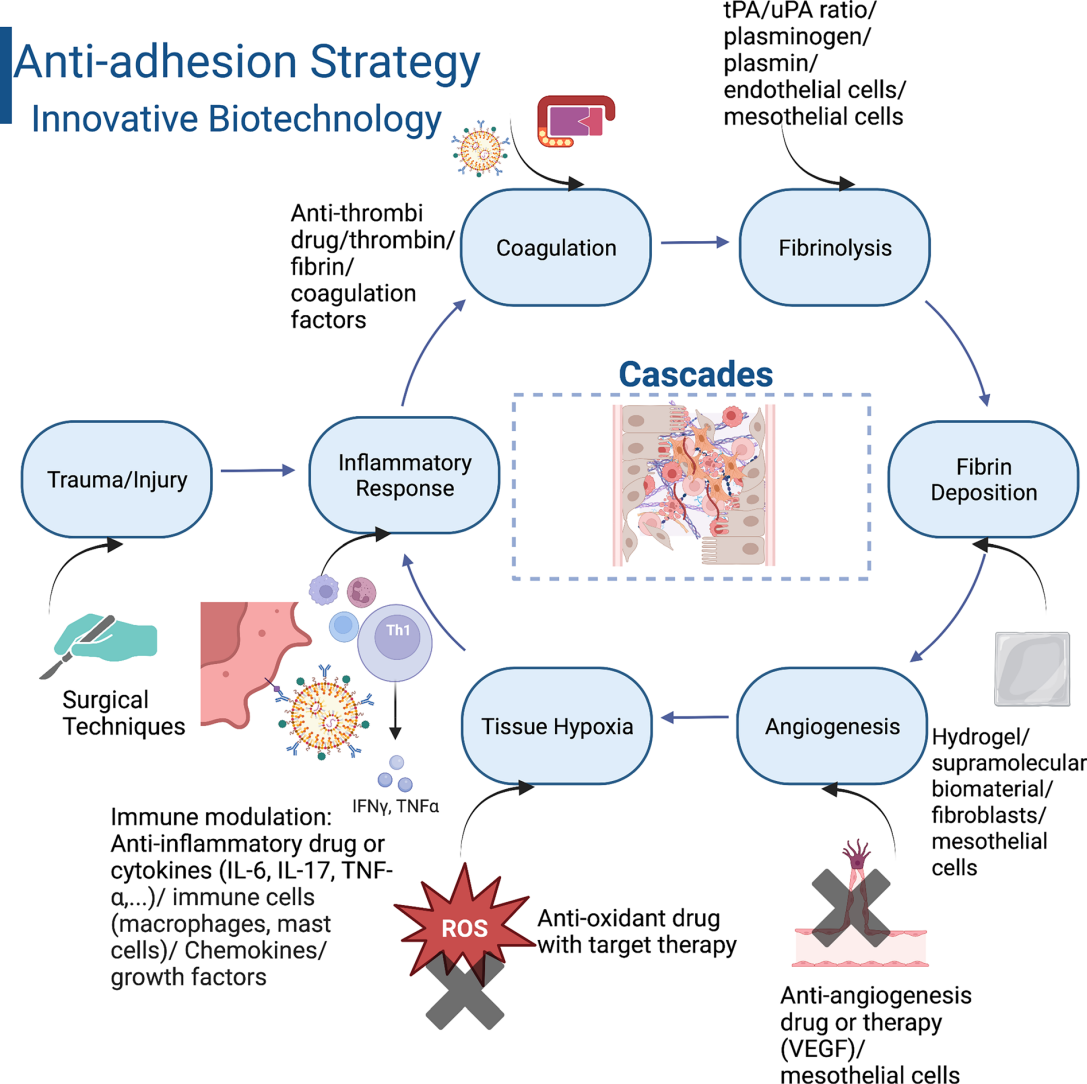
Anti‐dhesion future strategy.

From the current published literature, there are a significant number of studies that have introduced various biomaterials designed to prevent adhesion formation (Figure 5). These new biomaterials utilize a combination of physical, chemical, and/or biological properties to prevent the formation of adhesions. However, even with the creation of these innovative biomaterials, there has not been any significant clinical improvement in preventing or reducing the risk of adhesions. The only biomaterial which has made some level of advancement in impacting adhesion formation are physical barriers. For example, Stapleton et al. (2019) created a novel supramolecular polymeric hydrogel to reduce pericardial adhesion formation by allowing the hydrogel to completely encompass the heart while protecting it from the surrounding pericardium.^129^

Even with these encouraging findings, many studies have been focused on the prevention of abdominal adhesions which has its own set of challenges and problems that need to be overcome. One of these issues is the significant difference in size and total surface area within the abdominal cavity when considering the peritoneum and all internal abdominal organs, such as stomach, liver, gallbladder, pancreas, small intestine, and large intestine. Given the fact that the previously explored approach of physical barriers and chemical barriers has had limited success, it is suggested that an alternative focus on a biological mechanism may provide a solution to the issue. The integration of biological compounds such as interferons into biocompatible materials could provide a targeted way to prevent the adhesion formation by disrupting the normal cascades of events.

Another issue that needs to be addressed involves validating the anti‐adhesive properties of any biomaterial from an in‐vitro viewpoint, given that the in‐vivo environment cannot be accurately replicated in the laboratory. Currently, the major limitation of previous studies is the use of an in‐vitro assay is to assess biocompatibility requirements or to establish the appropriate material concentration in preparation for future in‐vivo studies. Fischer et al. (2020) have reported that they have created an in‐vitro beads induced adhesion assay to mimic the postsurgical adhesions. This is a novel approach for developing an in‐vitro adhesion formation model.^15^ Most of the studies listed in Table 2 used an in‐vivo approach as it provided a more standardized experimental model to study the combined impacts of inflammatory, coagulative, or fibroblastic activities on adhesion formation.

While anti‐adhesion membranes have been developed and used in clinical settings, there is still much research to be performed to fully understand the mechanisms of adhesion formation and develop effective solutions that prevent adhesion formation. There are also challenges associated with ensuring the safety and efficacy of anti‐adhesive biomaterials, particularly in long‐term applications. Ongoing research efforts are focused on addressing these challenges and developing novel biomedical devices that can prevent adhesion more safely and effectively.

## 4 CONCLUSION

In this paper, we have written an overview of the peritoneum anatomy, the pathophysiology of adhesion formation, as well as current strategies for preventing postsurgical adhesions. Based on our current understanding, postsurgical adhesions are triggered by trauma or tissue injury. Initial blood loss causes histamine release and increases the permeability of the vessels, followed by initiation of inflammatory response. At the same time, tissue hypoxia contributes to the mesothelial cells' stress and the release of the protein that generates inflammatory exudate. The wound‐healing process involves fibrinolysis and the coagulation cascade. Ischemia has been identified as an important factor that suppresses fibrinolytic activity and decreases fibroblasts infiltration resulting in the formation of persisting fibrin bands and adherent scar tissue.

Based on our current understanding of cellular and molecular adhesion mechanisms, different research studies have attempted to solve the issue by incorporating nanoparticles, anti‐inflammatory drugs, as well as fibrinolytic agents to intervene in the adhesion formation process. Several researchers have shown the positive results using animal studies either by applying nano‐micelles to deliver drugs or by using anti‐inflammatory drugs or fibrinolytic agents to modify or limit the adhesion cascades. However, due to the interdisciplinary nature of the research, no studies have designed the next‐generation anti‐adhesion barrier, nor have they been able to generate a clear understanding of the adhesion mechanism. Future studies will focus on utilizing a biomedical engineering platform to combine our current perspectives with the unique properties of advanced biomaterials using nanotechnology to develop a more effective and efficient anti‐adhesion barrier that can improve surgical outcomes.

## AUTHOR CONTRIBUTIONS

**Jiahui Chen:** Conceptualization (lead); Writing – original draft (lead); review and editing (lead); Visualization (lead). **Xiaoqi Tang:** Writing – original draft (equal). **Ziyu Wang:** Writing – original draft (supporting). **Arielle Perez:** Resources (equal). **Benjamin Yao:** Writing – review and editing (equal). **Ke Huang:** Writing – review and editing (equal). **Yang Zhang:** Funding acquisition (equal); writing – review and editing (equal). **Martin W King:** Funding acquisition (equal); resources (equal); supervision (equal); writing – original draft (equal); writing – review and editing (equal).

## FUNDING INFORMATION

We acknowledge the generous support from the National Institutes of Health (R21GM141675, R21GM141675) and the North Carolina Textile Foundation.

## CONFLICT OF INTEREST STATEMENT

The authors have no conflicts of interest to declare.

### PEER REVIEW

The peer review history for this article is available at https://www.webofscience.com/api/gateway/wos/peer‐review/10.1002/btm2.10565.

## Data Availability

Data sharing is not applicable to this article as no new data were created or analyzed in this study.
